# Radioimmunotherapy: A Specific Treatment Protocol for Cancer by Cytotoxic Radioisotopes Conjugated to Antibodies

**DOI:** 10.1155/2014/492061

**Published:** 2014-10-14

**Authors:** Hidekazu Kawashima

**Affiliations:** Department of Radiopharmaceutical Chemistry, Faculty of Pharmaceutical Sciences, Health Sciences University of Hokkaido, 1757 Kanazawa, Tobetsu-cho, Ishikari-gun, Hokkaido 061-0293, Japan

## Abstract

Radioimmunotherapy (RIT) represents a selective internal radiation therapy, that is, the use of radionuclides conjugated to tumor-directed monoclonal antibodies (including those fragments) or peptides. In a clinical field, two successful examples of this treatment protocol are currently extended by ^90^Y-ibritumomab tiuxetan (Zevalin) and ^131^I-tositumomab (Bexxar), both of which are anti-CD20 monoclonal antibodies coupled to cytotoxic radioisotopes and are approved for the treatment of non-Hodgkin lymphoma patients. In addition, some beneficial observations are obtained in preclinical studies targeting solid tumors. To date, in order to reduce the unnecessary exposure and to enhance the therapeutic efficacy, various biological, chemical, and treatment procedural improvements have been investigated in RIT. This review outlines the fundamentals of RIT and current knowledge of the preclinical/clinical trials for cancer treatment.

## 1. What Is Radioimmunotherapy (RIT)?

Antibodies (Abs) are glycoproteins secreted from plasma B cell and are used by immune system to identify and remove foreign pathogens such as bacteria and viruses. Because it is considered that Abs also have cytotoxic potency against some malignant tumor cells, the therapeutic efficacy in cancer has been examined. However, intact Abs are insufficient to improve patient survival rate dramatically. As a one approach to enhance the therapeutic response by using immunological technique, cytotoxic radioisotopes (*α*- or *β*-particle emitters) are conjugated to Abs or the fragments. This strategy is employed to deliver radioisotopes to the targeting tissue by appropriate vehicle. After the radiolabeled Abs bind to receptors/tumor antigens expressed on the surface of cancerous tissue, cells within an anatomic region of the *α*- or *β*-range will be killed.

In a clinical field, systemic radiotherapy using naked radioisotope (iodine-131: ^131^I) was first performed by Hertz to patient of Graves' disease in 1941 [1]. Then, investigations on the use of Abs coupled with adequate radioisotopes subsequently emerged in the early 1950s [2, 3]. Though direct radioiodinated Abs were mainly used in the initial clinical studies, progress in chelation chemistry has enabled the utilization of many therapeutic metal radioisotopes that possess inherent radiation properties. Various combinations of Abs and radioisotopes have been examined, which results in the adaptation in different clinical situations [4, 5]. RIT involves the application of radiolabeled monoclonal Abs (mAbs) to molecular targeted therapy [6]. Both the use of directly labeled mAbs and in vivo label of tumor-binding mAbs by conjugation-pretargeting method have been developed.

Irradiated cells absorb high amounts of energy in the form of photons or charged particles, which promote the direct macromolecular damage as well as the generation of reactive oxygen and/or nitrogen species [7]. Both free radicals and molecular oxygen damage DNA strand [8, 9], and the damage induces not only apoptosis [10] but also programmed necrosis [11]. Because the ranges in tissue of ionizing radiations are rather large compared with a typical cell size, uniform binding of the radioimmunoconjugates is not a prerequisite for its efficacy. In other words, adjacent cells not expressing the receptors/tumor antigens can also be killed by the physical cross-fire effect. This means continuous low-dose irradiation from radiolabeled Abs cause lethal effects on nearby normal cells. Moreover, it is reported that RITs also evoke the normalization of tumor vasculature [12], presumably owing to facilitation of immune cell migration towards the malignant lesions [13].

For therapy, therefore, *α*- or *β*-particle emitters are preferable. Vehicles coupled with radioisotopes emitting Auger electrons are also available; however, they need to be localized close to DNA due to the very short range of these radiations [14–16]. Simultaneous emission of *γ* (X) rays, which are suitable for imaging, will help measure pharmacokinetic parameters and calculate dosimetry of the radioimmunoconjugates.  Table 1 shows radioisotopes commonly used for RIT.

Among them, relatively well-studied and practical radioisotopes are the *β*-emitters ^131^I, yttrium-90 (^90^Y), and lutetium-177 (^177^Lu). Radioisotope to use is selected by consideration of those radiophysical properties (energy and half-life) as well as the labeling chemistry. For example, ^90^Y possesses a higher *β*-particle *E*_max⁡_ and a shorter half-life when compared with ^131^I. On the other hand, metal ^90^Y should be conjugated to Ab via chelating agent, whereas ^131^I can form a carbon-iodine bond directly. Lutetium-177 has radiophysical properties similar to ^131^I and radiolabeling chemistry similar to ^90^Y.

Investigations of RIT using *α*-particle emitters also have been developed. Because *α*-particle gives its energy to the surrounding molecules within a narrow range (<100 *μ*m, equivalent to a few cell diameters), it leads to high linear energy transfer (high LET) within the target and less bystander effect to nontarget tissues compared to Abs labeled with *β*-emitters. In addition to the high LET, which leads to the high relative biological effectiveness (RBE) [17], recent studies have shown that cytotoxic efficacy of *α*-particle is independent of the local oxygen concentration and cell cycle state [18]. Bismuth-213 (^213^Bi), astatine-211 (^211^At), and actinium-225 (^225^Ac) are well investigated in *α*-particle RIT [19–22].

Compared to external beam radiation therapy, one of the most potent advantages of RIT is the ability to attack not only the primary tumor but also lesions systemically metastasizing. In addition, targeted radiotherapy using specific vehicle agents is extremely valuable in cases of (1) residual micrometastatic lesions, (2) residual tumor margins after surgical resection, (3) tumors in the circulating blood including hematologic malignancy, and (4) malignancies that present as free-floating cells [23].

Brief data on current RITs provided in this review paper is summarized in Table 2.

## 2. Direct Method

The success of RIT depends on the selective accumulation of cytotoxic radioisotopes at affected areas. Fundamental properties required for vehicles against a particular biomarker are (1) high binding affinity to the intended target, (2) high specificity, (3) high tumor to background ratio, (4) high metabolic stability, and (5) low immunogenicity [24, 25]. From the viewpoint of those molecular characteristics, Abs have been considered as suitable agent for the delivery of therapeutic radioisotopes. Moreover, the development of hybridoma technology in 1975 allowed taking advantage of mAbs in RIT [26].

“Direct method” requires direct conjugation of cytotoxic radioisotopes to various antitumor mAbs (or their fragments) via an appropriate chelator and the single-step administration to patients. Consequently, many antigenic determinants (mostly on the cell surface) have been targeted by Abs. On the other hand, one of the most critical obstacles to achieve high background ratio in this application is the slow clearance of Abs from the blood and nontarget tissues due to their high molecular weight [27, 28]. Abs will disappear from plasma very slowly, which encourages higher tumor uptake; however, a longer duration is needed to reach the maximum tumor to normal radioactivity ratio. Radiation dose for treating patient increases time-dependently, which results in the exposure of radioactive bone marrow leading to the hematologic toxicity. Therefore, structural diversification of Abs has been attempted to improve the pharmacokinetic properties. Lower molecular weight fragments of conventional Abs including F(ab′)_2_, Fab or its multivalent conjugate, minibody, diabody, and single chain variable fragments (scFv) could be utilized, which retain the essential antigen binding properties and obtain more rapid clearance rates than intact mAbs. Those smaller types of constructs can traverse the vascular channels, resulting in a more rapid tumor uptake and a faster blood clearance than parental Abs [29, 30], possessing potencies to achieve superior tumor to background ratios. In general, however, affinities of small Ab forms to tumor antigen are lower than those of Abs, and, moreover, too fast blood clearance of peptides yields less time to interact with the target. Therefore, absolute tumor uptake for these constructs is lower than those of Abs. Further development of the engineered forms holding both favorable pharmacokinetics and tumor uptake is desired. Radiolabeled peptides targeting intended tumor can be available due to the preferable pharmacokinetics and low antigenicity. In this approach, control of the affinity (specific accumulation) of radiolabeled conjugates to tumor tissue is also important.

### 2.1. Hematological Cancers

It is reported that anticancer responses occur at relatively low radiation-absorbed doses (i.e., less than 10 Gy) in non-Hodgkin lymphoma (NHL) [31, 32]. ^90^Y-ibritumomab tiuxetan (Zevalin) and ^131^I-tositumomab (Bexxar) are the two FDA-approved radiolabeled anti-CD20 murine Abs that have been administered to patients with NHL [33, 34]. However, neither treatment is applied to patients with more than 25% bone marrow involvement because these patients might suffer from more severe hematologic toxicity. Several other radiolabeled Abs have been tested in hematological cancers. A ^131^I-labeled anti-CD20 mAb (^131^I-rituximab) [35–37] and an ^90^Y-labeled anti-CD22 mAb (^90^Y-epratuzumab tetraxetan) [32, 38, 39] are in advanced clinical trials.

Shorter-range radioisotopes (*α*-particle emitters) can be a better option for the treatment of patients with hematological cancer. In previous investigations, RIT using ^213^Bi-labeled anti-CD33 IgG was performed in myeloid leukemia [40, 41]; however, short physical half-life of ^213^Bi poses a problem for conjugate preparation. Thus, longer-lived emitters, such as ^211^At or ^225^Ac, which can emit four daughter *α*-particles during its decay, might be available. RITs using Auger-emitters, such as iodine-125 (^125^I), gallium-67 (^67^Ga), and indium-111 (^111^In), could be suitable for micrometastatic disease. Potential cytotoxicity of ^67^Ga-labeled anti-CD74 Ab was observed in a study using Raji B-lymphoma cells [42].

Due to the expression of these targeting antigens, normal B cells also have potencies to bind to the radiolabeled Abs. Thus, at low protein doses, the radioimmunoconjugates would be trapped to spleen rapidly, where a considerable number of B cells exist. To avoid this issue, unconjugated Ab is sometimes added to the system, which blocks the unfavorable distribution [43–45]. In addition, these Abs can enhance a tumor cell's sensitivity to radiation and chemotherapy [46–48], and thus, therapeutic responses would be achieved by a combination of the unconjugated Ab and targeted radiation.

### 2.2. Solid Cancers

RIT as a treatment protocol could be useful in the therapy for nonhematological cancers as well; however, convincing therapeutic outcomes have not been obtained yet in patients with solid cancer. One of the most obvious issues with RITs in solid cancers is that, unlike lymphoma, most of the Abs used are unable to affect tumor growth. To elicit clinical benefits for patients with advanced and/or disseminated solid cancers, several designs of the treatment are being undertaken: improvement of Ab uptake and enhancement of radiosensitization of cancer cells [49–51].

Here, some examples of RITs targeting solid cancer are shown.

#### 2.2.1. Colorectal Cancer

Owing to the early characterization and ubiquitous expression in colorectal cancer, carcinoembryonic antigen (CEA) [52] has been the most common target for RIT in this disease. cT84.66, a chimeric IgG against the A3 epitope of CEA, possesses highly selective affinity to cancer cells expressing CEA [53]. RIT using ^90^Y-cT84.66 was performed by Wong et al. [54, 55], with minor responses in tumor regression. Several other murine anti-CEA RIT agents have been evaluated, including ^131^I-NP-4 F(ab′)_2_, ^131^I-F6 F(ab′)_2_, ^131^I-A5B7, ^131^I-COL-1, and ^186^Re-NR-CO-02 F(ab′)_2_ [50, 56–60].

A33, one of the glycoproteins, is expressed homogeneously in more than 95% of all colorectal cancers [61] and thus the humanized Ab (huA33) has been developed. Preclinical studies using ^211^At-labeled huA33 indicate that the uptake in tumor was found to be specific to the presence of A33 antigen [62].

In a trial study performed by Liersch et al., patients having undergone liver resection for metastatic colorectal cancer were treated with ^131^I-labeled humanized anti-CEACAM5 IgG [63]. Median survival of patients having received RIT was significantly longer than that of control subjects [64].

#### 2.2.2. Breast Cancer/Ovarian Cancer

Trastuzumab is a humanized IgG mAb directed against the extracellular domain of the human epidermal growth factor receptor 2 (HER-2)/neu that is commonly overexpressed in breast, ovarian, and gastrointestinal tumors [65]. This Ab has been labeled with several radioisotopes such as ^90^Y [66] and ^111^In [67], for clinical study of breast cancer. Phase I study of intraperitoneal ^212^Pb-trastuzumab for patients with advanced ovarian cancer is also ongoing [68].

MUC-1, a mucin epitope, is commonly expressed on the surface of breast cancer cells. Schrier et al. reported on a phase I study using the murine Ab labeled with ^90^Y (^90^Y-MX-DTPA-BrE-3) and autologous stem cell rescue in patients with breast cancer [69]. One-half of the patients exhibited objective partial response to the therapy. To overcome the limitation of repeated dosing, an ^90^Y-labeled humanized Ab has also been evaluated for use with stem cell support [70].

Cell-surface sodium-dependent phosphate transport protein 2b, which is highly expressed on ovarian cancer cells, is recognized by the murine IgG MX35 [71, 72]. The potential usefulness of this Ab has been established in preclinical models, and then intraperitoneal administration of ^211^At-MX35 F(ab′)_2_ was undertaken to a phase I trial to determine the pharmacokinetics, dosimetry, and toxicity [73].

#### 2.2.3. Prostate Cancer

MUC-1, described above for its use in breast cancer, has also been shown to be upregulated in androgen-independent prostate cancer cells, making it a good target for RIT [74]. m170, a murine mAb, was labeled with ^90^Y, which was examined in patients with metastatic, androgen-independent prostate cancer [75]. Many patients who complain of pain at the entry of study reported a significant reduction in pain following therapy. A phase I study of ^90^Y-2IT-BAD-m170 combined with low-dose paclitaxel was also performed by the same group [76].

J591 is an IgG mAb against the extracellular domain of prostate-specific membrane antigen (PSMA). Several J591 constructs labeled with ^90^Y [77], ^177^Lu [78], and ^213^Bi [79] have also been evaluated in patients with prostate cancer.

## 3. Indirect Method

In “indirect method,” directly radiolabeled mAbs are not used; that is, mAbs and radioactive effector molecules are administered separately and they will be conjugated in vivo. This technique can improve target to nontarget ratio to achieve high imaging contrast and/or therapeutic efficacy. In a field of RIT, this strategy is referred to as pretargeted radioimmunotherapy (PRIT) and was developed to avoid the issues associated with the prolonged residence times of radiolabeled Ab in 1980s [80, 81].

**Figure 1:**
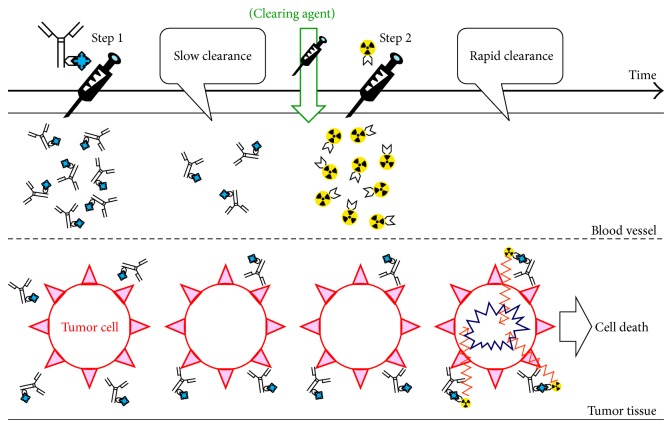
Schematic diagram of pretargeting approach.

PRIT is a technique that enables the Ab localization phase to be temporally separated from the radioisotope administration in the form of a small molecular hapten. This approach involves the sequential administration of (1) a bispecific mAb derivative (bs-mAb) capable of binding a tumor antigen and a chelate and (2) a small molecular weight radiolabeled effector species. The radiolabeled species is administered following a scheduled lag period to allow the bs-mAbs to accumulate to the target site and any residual bs-mAbs are cleared from the circulation. The bs-mAb is not radiolabeled directly, and thus no exposure would occur during “unlabeled” bs-mAbs localize to the tumor by themselves. In some cases, an additional “clearing molecule,” which removes unbound bs-mAb from the circulation, is administered prior to the radiolabeled effector. Consequently, improvement of tumor to background ratios has been achieved [82, 83]. Summarized scheme is shown in Figure 1.

A key to successful implementation of the pretargeting method consists of the high target specificity and affinity offered by bs-mAb and the superior pharmacokinetic characteristics of a low molecular weight compound. The small size and inert properties of the radiolabeled effector allow it to distribute easily in the fluidic volume and then to be eliminated rapidly, thereby decreasing the overall radiation burden to nontarget organs and tissues such as bone marrow [84]. Also, PRIT increases the dose rate to the cancer as compared with a RIT by using directly radiolabeled IgG that takes 1-2 days to reach maximum distribution.

To ensure the two parts (bs-mAb and radiolabeled effector) bind to each other strongly upon interaction at cancerous region, each must be suitably modified with complementary reactive species. One of the approaches is based on avidin or streptavidin in conjunction with biotin in a variety of configurations [85]. Avidins could bind as many as four biotin molecules with very high binding constant (10^−15 ^M), and, thus, some avidin/biotin-based PRIT were examined clinically [86–88]. In this protocol, clearing agent was injected to remove residual streptavidinated Abs from the blood. The primary issue with PRIT depending on avidin (streptavidin) is the immunogenicity of these foreign proteins [89, 90].

Conjugation of radioisotopes to bs-mAb is controlled by another constituent. The approach has been utilized with a bs-mAb to histamine-succinyl-glycine (HSG) [91, 92]. By joining two haptens via a short peptide, uptake and retention of the radiolabeled effector (divalent hapten-peptide) would be enhanced locally within the cancer compared with those of the monovalent form [93–95]. Di-HSG-peptide structures have been developed for binding several radioisotopes, including ^90^Y and ^99m^Tc [96, 97]. Other methods employ complementary synthetic low immunogenic DNA analogs, morpholinos, as bridging agents [98, 99].

### 3.1. PRIT for Hematological Cancers

In preclinical studies, pretargeting method showed better responses with much less hematologic toxicity and thus represents a significant improvement over the RIA using anti-CD20 IgG agents radiolabeled directly [100].

Among the hematological cancers, NHL therapy has been examined in detail by pretargeting. In conventional NHL model mice, PRIT with a tri-Fab fragment followed by ^90^Y-labeled effector led to dramatic cure rates compared to RIT with direct ^90^Y-veltuzumab, which is a radiolabeled anti-CD20 Ab [101]. Similar therapeutic responses were also achieved using the streptavidin-biotin format of PRIT in the Ramos lymphoma model, using a combination of anti-CD20 Ab-streptavidin fusion protein 1F5(scFv)_4_SA and ^90^Y-DOTA-biotin [102]. Pagel et al. compared therapeutic efficacy between direct RIT and PRIT in xenograft models of lymphoma using CD20, CD22, and MHC class II cell surface receptor (HLA-DR) as the targets. In this study, PRIT by the streptavidin-biotin conjugation showed higher therapeutic indices and superior tumor regression [103].

### 3.2. PRIT for Solid Cancers

A phase II trial was examined with ^90^Y-DOTA-biotin pretargeted with a NR-LU-10, an anti-Ep-CAM (epithelial glycoprotein-2) IgG-streptavidin conjugate in advanced colorectal cancer [87]; however, no significant responses were observed. More recently, a recombinant protein of streptavidin with four CC49 (anti-tumor-associated glycoprotein 72: anti-TAG-72) single chains and ^90^Y-labeled biotin pair has been tested in patients with gastrointestinal malignancy [104].

In a field of brain tumor, patients with grade III glioma and glioblastoma were pretargeted with ^90^Y-labeled biotin, resulting in a significant extension of survival in the PRIT subjects [105].

## 4. Concluding Remarks

Because Ab-based targeted radiation is considered to mediate direct cytotoxic effects, RIT (PRIT) could provide us with opportunities for safer and more efficient cancer treatment. Indeed, these techniques have been extensively used as conventional anticancer strategies. Especially, RIT (PRIT) has been effective in hematological cancers. There are also developments of new immunostimulant for lymphoma that is combined with RIT (PRIT), which can enhance the overall therapeutic response.

On the contrary, responses to RIT (PRIT) are generally low in solid cancer and it might be due to the unconventional microenvironments. Oxygen concentrations less than 0.02% decrease the vulnerability of cancer cells to ionizing radiation [106], and even milder hypoxia produces a substantial level of resistance to irradiation [107]. Strategies to radiosensitize the lesions by means of an increased supply of oxygen or treatment of nitroimidazole analog [108] would help enhance the efficacy of RIT (PRIT).

RIT (PRIT) is a valuable treatment modality which can detect and quantify the accumulation of therapeutic agents readily. Thus, molecular imaging approach can be adapted to select patients, to decide the treatment strategy, and to assess the therapeutic benefit. Developments of novel Ab-based targeted therapeutics and the combination with other interventions should support cancer therapy in future.

## Figures and Tables

**Table 1:** Radioisotopes used in RIT.

Radioisotope	Energy_max⁡_ (MeV)	Range	Half-life
*β*-Particle emitter			
^ 67^Cu	0.58	2.1 mm	2.6 d
^ 90^Y	2.28	12.0 mm	2.7 d
^ 131^I	0.61	2.0 mm	8.0 d
^ 177^Lu	0.50	1.5 mm	6.7 d
^ 186^Re	1.07	4.5 mm	3.7 d
^ 188^Re	2.12	10.4 mm	16.9 hr

*α*-Particle emitter			
^ 211^At	6.8	80 *μ*m	7.2 hr
^ 213^Bi	8.3	84 *μ*m	46 min
^ 225^Ac	6.0~8.0	60~90 *μ*m	10.0 d

Auger-electron emitter			
^ 125^I		2~500 nm	60.5 d

**Table 2:** Anticancer RITs reported in this century.

Cancer	Target molecule	mAb	Radioisotope	Subject	Reference
Direct method					
Non-Hodgkin lymphoma	CD20	Ibritumomab	Y-90	Human (in clinical use)	[33]
		Tositumomab	I-131	Human (in clinical use)	[31, 33, 34]
		Rituximab	I-131	Human (phase II)	[35–37]
	CD22	Epratuzumab	Y-90	Human (phase II)	[32, 38, 39]
Myeloid leukemia	CD33	Lintuzumab	Bi-213	Human (phase II)	[40, 41]
Raji B-lymphoma	CD74	L243	Ga-67	Cell	[42]
Colorectal cancer	Carcinoembryonic antigen (CEA)	cT84.66	Y-90	Human (phase I)	[54, 55]
	A33 glycoprotein	huA33	At-211	Mouse xenograft model	[62]
Colorectal cancer (liver metastases)	CEA	F6 F(ab′)_2_	I-131	Human (phase II)	[58]
	CEA-related cell adhesion molecule	Labetuzumab	I-131	Human (phase II)	[63, 64]
Gastrointestinal cancer	CEA	A5B7	I-131	Human (phase I)	[48]
Breast cancer	HER-2	Trastuzumab	Y-90	Mouse xenograft model	[66]
			Pb-212	Human (phase I)	[68]
		NLS-trastuzumab	In-111	Cell	[67]
Ovarian cancer	Na-dependent phosphate transporter	MX35 F(ab′)_2_	At-211	Human (phase I)	[73]
Prostate cancer	MUC-1	m170	Y-90	Human (phase I)	[75, 76]
		J591	Y-90	Human (phase I)	[77]
			Lu-177	Human (phase I)	[78]
			Bi-213	Mouse xenograft model	[79]
Multiple myeloma	CD138	Anti CD138 Ab	Bi-213	Mouse xenograft model	[20]

Indirect method					
Non-Hodgkin lymphoma	CD20	TF4 (HSG)	Y-90	Mouse xenograft model	[100, 101]
		1F5(scFv)_4_ (streptavidin)	Y-90	Mouse xenograft model	[102]
	CD20, CD22, HLA-DR	Corresponding Abs (streptavidin)	Y-90	Mouse xenograft model	[103]
Colon cancer	CEA	hBS14 (HSG)	Y-90	Mouse xenograft model	[96]
		MN14 (MORF)	Re-188	Mouse xenograft model	[98]
	Ep-CAM	NR-LU-10 (HSG)	Y-90	Human (phase II)	[87]
Gastrointestinal cancer	TAG-72	CC49-(scFv)_4_ (streptavidin)	Y-90	Human (phase I)	[104]
Glioma	Tenascin	BC4 (biotin)	Y-90	Human (phase II)	[105]
